# Protective and Enhancing HLA Alleles, HLA-DRB1*0901 and HLA-A*24, for Severe Forms of Dengue Virus Infection, Dengue Hemorrhagic Fever and Dengue Shock Syndrome

**DOI:** 10.1371/journal.pntd.0000304

**Published:** 2008-10-01

**Authors:** Nguyen Thi Phuong Lan, Mihoko Kikuchi, Vu Thi Que Huong, Do Quang Ha, Tran Thi Thuy, Vo Dinh Tham, Ha Manh Tuan, Vo Van Tuong, Cao Thi Phi Nga, Tran Van Dat, Toshifumi Oyama, Kouichi Morita, Michio Yasunami, Kenji Hirayama

**Affiliations:** 1 Department of Immunogenetics, Institute of Tropical Medicine (NEKKEN), Nagasaki University, Japan; 2 Center of International Collaborative Research, Nagasaki University, Japan; 3 Laboratory of Arbovirus, Pasteur Institute in Ho Chi Minh City, Vietnam; 4 Children's Hospital No.2, Ho Chi Minh City, Vietnam; 5 Center for Preventive Medicine, Vinh Long Province, Vietnam; 6 Global COE program, Nagasaki University, Japan; 7 Department of Virology, Institute of Tropical Medicine (NEKKEN), Nagasaki University, Japan; University of California Berkeley, United States of America

## Abstract

**Background:**

Dengue virus (DV) infection is one of the most important mosquito-borne diseases in the tropics. Recently, the severe forms, dengue hemorrhagic fever (DHF) and dengue shock syndrome (DSS), have become the leading cause of death among children in Southern Vietnam. Protective and/or pathogenic T cell immunity is supposed to be important in the pathogenesis of DHF and DSS.

**Methodology/Principal Findings:**

To identify HLA alleles controlling T cell immunity against dengue virus (DV), we performed a hospital-based case control study at Children's Hospital No.2, Ho Chi Minh City (HCMC), and Vinh Long Province Hospital (VL) in Southern Vietnam from 2002 to 2005. A total of 211 and 418 patients with DHF and DSS, respectively, diagnosed according to the World Health Organization (WHO) criteria, were analyzed for their characteristic HLA-A, -B and -DRB1 alleles. Four hundred fifty healthy children (250 from HCMC and 200 from VL) of the same Kinh ethnicity were also analyzed as population background. In HLA class I, frequency of the HLA-A*24 showed increased tendency in both DHF and DSS patients, which reproduced a previous study. The frequency of A*24 with histidine at codon 70 (A*2402/03/10), based on main anchor binding site specificity analysis in DSS and DHF patients, was significantly higher than that in the population background groups (HCMC 02-03 DSS: OR = 1.89, P = 0.008, DHF: OR = 1.75, P = 0.033; VL 02-03 DSS: OR = 1.70, P = 0.03, DHF: OR = 1.46, P = 0.38; VL 04-05 DSS: OR = 2.09, P = 0.0075, DHF: OR = 2.02, P = 0.038). In HLA class II, the HLA-DRB1*0901 frequency was significantly decreased in secondary infection of DSS in VL 04-05 (OR = 0.35, P = 0.0025, Pc = 0.03). Moreover, the frequency of HLA-DRB1*0901 in particular was significantly decreased in DSS when compared with DHF in DEN-2 infection (P = 0.02).

**Conclusion:**

This study improves our understanding of the risk of HLA-class I for severe outcome of DV infection in the light of peptide anchor binding site and provides novel evidence that HLA-class II may control disease severity (DHF to DSS) in DV infection.

## Author Summary

Dengue has become one of the most common viral diseases transmitted by infected mosquitoes (with any of the four dengue virus serotypes: DEN-1, -2, -3, or -4). It may present as asymptomatic or illness, ranging from mild to severe disease. Recently, the severe forms, dengue hemorrhagic fever (DHF) and dengue shock syndrome (DSS), have become the leading cause of death among children in Southern Vietnam. The pathogenesis of DHF/DSS, however, is not yet completely understood. The immune response, virus virulence, and host genetic background are considered to be risk factors contributing to disease severity. Human leucocyte antigens (HLA) expressed on the cell surface function as antigen presenting molecules and those polymorphism can change individuals' immune response. We investigated the HLA-A, -B (class I), and -DRB1 (class II) polymorphism in Vietnamese children with different severity (DHF/DSS) by a hospital-based case-control study. The study showed persons carrying HLA-A*2402/03/10 are about 2 times more likely to have severe dengue infection than others. On the other hand, HLA-DRB1*0901 persons are less likely to develop DSS with DEN-2 virus infection. These results clearly demonstrated that HLA controlled the susceptibility to severe forms of DV infection.

## Introduction

Dengue virus (DV) infection has become one of the most important mosquito-borne diseases in the tropics [1]. An estimated 50 million people worldwide are infected with DV each year [2]. There are two principal severe forms of DV infection, namely dengue hemorrhagic fever (DHF) and dengue shock syndrome (DSS). Other severe forms such as encephalitis and spastic tetraparesis occur less frequently [3]. Children younger than 15 years of age account for 90% of the severe cases in Southeast Asian countries [4] where the DHF/DSS incidence has increased about 5 fold more rapidly since 1980 than in the previous 30 years [5]. These severe forms of DV infection have become the leading cause of death among children in Southern Vietnam [6].

Infection by any of the 4 serotypes of DV, DEN-1, -2, -3, and -4, may result in no symptoms, dengue fever (DF, without any serious complications), DHF or DSS. DF is characterized by high fever; severe headache; retro-orbital, muscle, bone or joint pains; and rash. DHF is characterized by the development of plasma leakage near the time of defervescence, thrombocytopenia, coagulation abnormalities, and hemorrhage. Hypotension with massive hemorrhage and plasma leakage characterize the most serious form of DHF called DSS [3]. DHF in this study corresponds to a milder form of DHF that WHO criteria define as grade I and II, and DSS to a grade III and IV DHF. The four criteria in the WHO case definition for DHF (fever, hemorrhage, thrombocytopenia and plasma leakage) were claimed not so practical because serious cases sometimes lack one or more of them, particularly hemorrhage and thrombocytopenia. Instead the Integrated Management of Childhood Illness (IMCI) has recently proposed the use of the terms “dengue” and “severe dengue” for the symptomatic patients with no emphasis on those two signs [7]

The pathogenesis of DHF/DSS is not yet completely understood. Host immunity has been extensively analyzed, including studies of antibody-dependent enhancement (ADE) [8], complement activation [8], anti-platelet antibodies [8], suppressed Th1/predominant Th2 functions [4] and the production of cytokines [9],[10] and cytotoxic factors [4]. Virus factor has also been studied. DEN-2 viral strains were associated with DHF/DSS in Thailand and Vietnam; DEN-3 strains have been predominant in the DHF/DSS in Sri Lanka, Indonesia and India; while in Latin America, DEN-2,-3,-4 have all been commonly associated with severe disease [8],[11]. In addition to those, still unidentified variations in immune response, virus virulence, and host genetic background are considered to be risk factors contributing to disease development [1].

Several genetic factors potentially influencing the severity of dengue infection, such as vitamin D receptor (VDR), Fcγ receptor II (FcγRII), interleukin-4 (IL-4), interleukin-1 receptor antagonist (IL-1RA), and mannose-binding lectin (MBL) were investigated in Vietnamese [12]. Among them, the T allele at position 352 of the VDR gene was associated with resistance to DSS as well as homozygotes for the arginine variant at position 131 of the FcγRII gene were shown to be protective against DSS. Additionally, the G allele at position -336 of DC-SIGN (dendritic cell-specific ICAM-3 grabbing nonintegrin) was reported to be protective against DF among the Thai population [13].

Dengue virus, like other members of the *Flaviviridae* family, increases the expression of HLA class I and II molecules on infected cells [14]. HLA-controlled immune response may be responsible for the immunopathology of DV infection [14]. The host HLA allele profile influenced the reactivity of DV-specific T cells [15],[16] however, there have been only a few informative studies on HLA association with the severity of DV infection [17],[18]. In the present study, we investigated the HLA-A, HLA-B, and HLA-DRB1 polymorphisms in the Vietnamese population and the association of HLA alleles with two different clinical forms of severe DV infection during the years 2002–2005.

## Materials and Methods

### Study subjects

The study was performed at two hospitals, the Children's Hospital No. 2 in Ho Chi Minh City (HCMC) and the Center for Preventive Medicine in the Vinh Long Province (VL) of the Mekong Delta. The enrolment was consecutive sequence of children hospitalized at each hospital. The inclusion criteria at the entry point in the hospital were age 6 months to 15 years old, Kinh race, and unrelatedness. The subjects enrolled initially were 325, 356 and 403 in HCMC 2002–2003 (HCMC 02-03), VL 2002-2003 (VL 02-03) and VL 2004–2005 (VL 04-05), respectively. DV infection of the patients was diagnosed by clinical symptoms and history at the admission time. After hospitalization, the patients were diagnosed by the well established serology as described below.

WHO classification criteria [3] was applied after a review of case report form (CRF) of each patient. The DHF classification required fever or a history of acute fever, bleeding manifestation, and signs of plasma leakage which include hemoconcentration, ascites, or pleural effusion with evidence of thrombocytopenia. Hemoconcentration (more than 20% increase in Hct) was evaluated by estimating the percent increase in the Hct by comparing two values recorded at 2 different timings: the maximum value in defervescence day and the value before discharge. The ascites, or pleural effusion were detected by echography or X-ray. The DSS classification required DHF manifestation plus evidence of clinical hypovolemic shock (tachycardia and narrow pulse pressure (<20 mmHg))

It was previously reported that the sensitivity of WHO criteria for DSS in Vietnam was 82%, mainly due to the lack of evidence for thrombocytopenia [19]. Therefore, we basically followed the WHO criteria but included patients lacking the significant reduction of platelet count, which accounted for no more than 11% of all DHF/DSS cases. Our classification met the requirement of the simplified classification system of IMCI, which is based on plasma leakage as a hallmark of severe dengue (DHF/DSS) [7].

Healthy unrelated school children living in HCMC and VL (250 and 200 subjects respectively) who had no symptoms of dengue virus infection were collected as a background population control group for the genetic study. In these control groups, 13 cases (5.2%) in HCMC and 16 cases (8.0%) in VL were seropositive for IgM ELISA to DV, indicating recent asymptomatic DV infection.

This study was approved by the institutional ethical review committees of the Institute of Tropical Medicine, Nagasaki University, and the Pasteur Institute in Ho Chi Minh City. Informed consent was obtained from the parents or legal guardians of the subjects upon enrollment.

### Sample collection and serological diagnosis

Blood samples were collected from patients with suspected dengue infection at the time of enrolment to the study and in the convalescent phase prior to discharge from the hospital. Plasma samples were used for titration of anti-DV IgM and IgG antibodies, virus isolation, and RT-PCR for determination of viral serotype; buffy coat samples were used to extract genomic DNA by using the QIAamp DNA blood kit (Qiagen, Hilden, Germany). DV infection was defined by previously established serologic criteria for IgM/IgG ELISAs to DV (DEN 1–4) and Japanese encephalitis virus (kit of Pasteur Institute HCMC) in paired sera, collected at least three days interval [20]. IgM and IgG ELISAs were considered positive if the ratio of optical density (OD) of test sera to OD of negative control sera was ≥2.3 [3]. The cases were diagnosed as secondary infection when DV IgM-to-IgG ratio was <1.8 [20].

### Dengue virus serotyping

Acute plasma samples were inoculated into C6/36 (*Aedes albopictus*) cells, the virus was then obtained and the dengue virus serotype was identified using the direct or indirect fluorescent antibody technique monoclonal antibodies supplied by the Centers for Disease Control and Prevention (Fort Collins, CO, USA) [21]. Viral RNA was also extracted from the same acute plasma samples with the QIAamp Viral RNA Mini Kit (Qiagen, Hilden, Germany) for detection of DV and confirmation of its serotype as described [21]. Briefly, cDNA from the DV genome RNA was amplified with the Ready-to-go RT-PCR test kit (Amersham, MA, USA) using consensus primer set (D1 and D2) [22]. Then, the serotype was determined by semi-nested PCR to amplify serotype-specific fragments from the regions encoding the capsid and membrane proteins of DV using the specific primer sets (TS1, TS2, TS3, and TS4) [22].

### HLA typing

HLA-A, -B and -DRB1 were typed by using LABType SSO class I A and B and class II DRB1 locus kits (One Lambda, CA, USA) and the Luminex LX 100 IS system (Hitachi, Japan). IS 2.3 typing software (Luminex Corporation, TX, USA) was used to determine the genotype. The presence of alleles of interest were confirmed by PCR with sequence-specific primers (PCR-SSP) [23] (HLA-A*24) and sequencing-based typing (HLA-DRB1*0901) [according to the standard protocol 10-B of the 13th International Histoconpatibility Workshop (http://www.ihwg.org/protocols/sbt/sbtprot.htm)]. HLA-A, HLA-B, and HLA-DRB1 genotype distributions were checked with Hardy-Weinberg equilibrium.

### Statistical analysis

The frequencies of individuals carrying particular alleles (phenotype frequencies) were compared between categories of the case group (DHF or DSS) and the population background group. The difference in frequency was evaluated by the odds ratio (OR) with Fisher exact 95% confidence intervals (95% CI), and Exact Fisher two sided P using StatsDirect statistical software, version 2.6.4. The locus-wise correction of the P value (Pc) for multiple tests was made by multiplying the P value by the number of the major alleles whose phenotype frequencies were more than 5% in either the patient or population background group. The correction factors (CF) were 8 for HLA-A, 13 for HLA-B and 12 for HLA-DRB1. Difference yielding Pc values less than 0.05 was considered statistically significant.

## Results/Discussion

### Clinical features of the patients

Demographic and clinical features of the study subjects are shown in Table 1. An important clinical findings in this study was hepatomegaly which was clearly more prevalent in DSS than in DHF patients (in HCMC 02-03: 90.8% vs. 47.9%, in VL 02-03: 98.8% vs. 88.6%, and in VL 04-05: 93.8% vs. 78.0%, ORs above 4, footnote b of Table 1). There was substantial evidence of hepatomegaly in Asian patients [24],[25] as well as in South American patients [26] with DHF/DSS. Our results therefore agree with the previous study by Pham *et al.* which propose hepatomegaly as one of the clinical predictors of DSS in children [27].

**Table 1:** Study subjects

	Ho Chi Minh City (HCMC)			Vinh Long province (VL)				
	HCMC 02-03			VL02-03		VL04-05		
	DSS	DHF	Controls	DSS	DHF	DSS	DHF	Control
	n = 152	n = 117	n = 250	n = 170	n = 35	n = 96	n = 59	n = 200
Mean age (years)±SD	9.1±3.2	10.6±3.2	7.8±4.4	9.9±3.0	8.6±4.2	8.6±3.4	8.8±4.2	10.9±2.2
Male (%)^a^	80 (52.6)	70 (59.8)	143 (57.0)	78 (45.9)	16 (45.7)	37 (38.5)	36 (61.0)	110 (55.0)
Hepatomegaly (%) b	138 (90.8)	56 (47.9)	nd	168 (98.8)	31 (88.6)	90 (93.8)	46 (78.0)	nd
Percent increase in Hct (%)±SD	36.6±16.5	27.4±9.8	nd	34.5±20.9	31.2±17.2	26±19.9	23.3±7.7	nd
Secondary infection (%)^c^	81 (53.3)	50 (42.7)	nd	95 (55.9)	13 (37.1)	80 (83.3)	46 (78.0)	nd

DHF: dengue hemorrhagic fever, DSS: dengue shock syndrome, SD: standard deviation, nd: not done, P: Fisher's exact test, Hct: hematocrit.

a Male among DSS patients in VL04-05 vs. VL controls: P = 0.008

b Hepatomegaly among DSS patients vs. DHF patients, in HCMC 02-03: OR = 10.74, P<0.0001, in VL 02-03: OR = 10.84, P = 0.008, in VL 04-05:OR = 4.24, P = 0.005

c Secondary infection in DSS VL04-05 vs. HCMC 02-03: P<0.0001, VL04-05 vs. VL02-03: P<0.0001 and in DHF VL04-05 vs. HCMC 02-03: P<0.0001, VL04-05 vs. VL02-03: P = 0.0001

Another point of note was concerning the issue of significance of secondary infection of DV in the pathogenesis of severe cases. Although secondary infection has been reported to confer most of the risk of disease severity, it is also known that patients without evidence of secondary infection substantially contribute to severe cases in Taiwan [28] and Thailand [29]. Our study is similar to that study in Thailand 2003 [29], where the patients with high IgM/IgG ratio consist of nearly 60% of DSS and 40% of DHF. The data still support the notion of disease risk associated with secondary infection (footnote c of Table 1).

It is worth to mention that four percents of our patients were under 1 year of age and all had primary DV infection. The presence of non neutralizing or low level of neutralizing maternal antibodies may play a role in the development of severe disease. Halstead *et al.* pointed out that infants are a high risk group for DHF/DSS, and need to be studied to further understand primary dengue infection [30].

### DV serotypes and disease severity

Virus serotype was tested on 388 patients whose blood samples collected within five days after the onset of symptoms, mostly on days 4 and 5. As shown in Table 2, DV was identified in 87 DHF/DSS patients, which gave a detection rate of 22.4%. This was far lower than many other studies but consistent with a previous observation that the sensitivity to detect DV is dulled in the present of neutralizing antibody [20]. Similar low detection rate was reported in Bangladesh study in 2002 [31], where virus serotype could only be identified in 8% of samples.

**Table 2:** Dengue virus serotypes and disease severity (2002–05)

	Number of cases (%)				DSS vs. DHF	
	DSS (n = 43)		DHF (n = 44)		OR (95% CI)	P
DEN-1	10	(23.3)	6	(13.6)	1.90 (0.56–7.11)	0.3
DEN-2	30	(70.0)	20	(45.5)	2.77 (1.05–7.37)	0.03
DEN-3	3	(7.0)	10	(22.7)	0.26 (0.04–1.11)	0.07
DEN-4	0	(0.0)	8	(18.2)	0.05 (0.003–0.88)	0.006

DHF: dengue hemorrhagic fever, DSS: dengue shock syndrome

OR: Odds ratio, 95% CI: Fisher exact 95% confidence intervals, P: Fisher's exact test

DEN-2 constituted the majority of the total isolates, 50 (57.5%) followed by 16 DEN-1 (18.4%), 13 DEN-3 (14.9%) and 8 DEN-4 (9.2%). The correlation between DEN-2 infection and the severe clinical forms (Table 2) provided additional proof for higher virulence of this serotype because previous study reported that patients with DEN-2 had a larger pleural effusion index than those infected by other virus serotypes [32].

### Phenotype frequencies of HLA-A, HLA-B and HLA-DRB1

We identified 16 HLA-A alleles, 47 HLA-B alleles and 36 HLA-DRB1 alleles in the study subjects. Major alleles (phenotype frequencies having more than 5% in either the patient or healthy background groups) are shown in Table 3 and Table S1, accounted for about 80–90% of the total phenotypes. We analyzed only these major alleles for the evaluation of the risk of disease severity because rare alleles would have little impact on population risk. There were no significant difference in phenotype frequencies of the major alleles in the 2 population background groups (Table S1), and these data were compatible with data from a previous study [17] on the same Kinh ethnic in Southern Vietnam.

**Table 3:** Major alleles of HLA-A, -B, -DRB1 in Vietnamese

HLA	Allele	Total
HLA-A	*01, *02, *11, *24, *26, *29, *30, *33	8
HLA-B	*07, *13, *15, *27, *35, *38, *40, *44, *46, *51, *55, *57, *58	13
HLA-DRB1	*0301, *0403, *0405, *0701, *0803, *0901, *1001, *1202, *1401, *1501, *1502 *1602	12

### HLA-A*24 was positively associated with DSS or DHF

As shown in Table 4 and 5, HLA-A*24 increased in frequency among DHF and DSS patients when compared with the control in all 3 sample groups, HCMC 02-03 (DSS: OR = 1.62, P = 0.04, Pc = 0.3; DHF: OR = 1.35, P = 0.26), VL 02-03 (DSS: OR = 1.51, P = 0.08; DHF: OR = 1.34, P = 0.4), and VL 04-05 (DSS: OR = 1.80, P = 0.029, Pc = 0.2; DHF: OR = 2.71, P = 0.0019, Pc = 0.02).

**Table 4:** Phenotype frequencies of HLA-A*24 and HLA-DRB1*0901 in primary and secondary infection and population background groups

Phenotype			HCMC						VL									
			HCMC 02-03						VL 02-03				VL 04-05					
			DSS		DHF		Control		DSS		DHF		DSS		DHF		Control	
			No.	(%)	No.	(%)	No.	(%)	No.	(%)	No.	(%)	No.	(%)	No.	(%)	No.	(%)
1. HLA-A*24																		
	1.1	A*24	53	(34.9)	36	(30.8)	62	(24.8)	57	(33.5)	11	(31.4)	36	(37.5)	28	(47.5)	50	(25.0)
	1.2	A*24 Primary infection	28	(39.4)	25	(37.3)	62	(24.8)	28	(37.3)	8	(36.4)	3	(18.8)	7	(58.3)	50	(25.0)
	1.3	A*24 Secondary infection	25	(30.9)	11	(22.0)	62	(24.8)	29	(30.5)	3	(23.0)	33	(41.3)	21	(45.7)	50	(25.0)
2. HLA- DRB1*0901																		
	2.1	DRB1*0901	33	(21.7)	31	(26.5)	61	(24.4)	35	(20.6)	6	(17.1)	14	(14.6)	15	(25.4)	63	(31.5)
	2.2	DRB1*0901 Primary infection	15	(21.1)	18	(26.9)	61	(24.4)	17	(22.7)	2	(9.1)	3	(18.8)	4	(33.3)	63	(31.5)
	2.3	DRB1*0901 Secondary infection	18	(22.2)	13	(26.0)	61	(24.4)	18	(18.9)	4	(30.8)	11	(13.8)	11	(23.9)	63	(31.5)

DHF: dengue hemorrhagic fever, DSS: dengue shock syndrome

**Table 5:** Association of HLA-A*24, -DRB1*0901 allele with severe forms

			HCMC			VL					
			HCMC 02-03			VL 02-03			VL 04-05		
			OR (95% CI)	P	Pc	OR (95% CI)	P	Pc	OR (95% CI)	P	Pc
1. HLA- A*24											
	1.1	DSS vs. control	1.62 (1.02–2.58)	0.04	0.3	1.51 (0.94–2.44)	0.084	-	1.80 (1.03–3.13)	0.029	0.2
	1.2	DSS with primary infection vs. controls	1.97 (1.08–3.56)	0.024	0.2	2.38 (1.30–4.32)	0.0032	0.03	0.69 (0.12–2.67)	0.77	-
	1.3	DSS with secondary infection vs. controls	1.35 (0.74–2.42)	0.3	-	1.76 (0.99–3.09)	0.045	0.36	2.11 (1.17–3.77)	0.009	0.07
	1.4	DHF vs. control	1.35 (0.80–2.25)	0.26	-	1.34 (0.57–3.16)	0.4	-	2.71 (1.41–5.16)	0.0019	0.02
	1.5	DHF with primary infection vs. controls	1.13 (0.63–2.00)	0.67	-	2.29 (0.78–6.20)	0.1	-	4.2 (1.08–17.43)	0.018	0.1
	1.6	DHF with secondary infection vs. controls	0.86 (0.37–1.83)	0.7	-	1.20 (0.20–4.89)	0.73	-	2.52 (1.22–5.13)	0.007	0.056
	1.7	DSS vs. DHF	1.20 (0.70–2.09)	0.51	-	1.1 (0.48–2.67)	1	-	0.66 (0.33–1.35)	0.24	-
2. HLA- DRB1*0901											
	2.1	DSS vs. control	0.86 (0.51–1.42)	0.63	-	0.56 (0.34–0.93)	0.018	0.2	0.37 (0.18–0.72)	0.0018	0.02
	2.2	DSS with primary infection vs. controls	0.83 (0.41–1.62)	0.64	-	0.64 (0.32–1.22)	0.18	-	0.50 (0.09–1.92)	0.4	-
	2.3	DSS with secondary infection vs. controls	0.89 (0.46–1.66)	0.77	-	0.51 (0.26–0.95)	0.026	0.3	0.35 (0.16–0.72)	0.0025	0.03
	2.4	DHF vs. control	1.12 (0.65–1.90)	0.69	-	0.45 (0.15–1.18)	0.10	-	0.74 (0.36–1.48)	0.42	-
	2.5	DHF with primary infection vs. controls	1.14 (0.58–2.17)	0.75	-	0.22 (0.02–0.95)	0.027	0.3	1.09 (0.23–4.24)	1	-
	2.6	DHF with secondary infection vs. controls	1.09 (0.50–2.26)	0.86	-	0.97 (0.21–3.63)	1	-	0.68 (0.29–1.49)	0.37	-
	2.7	DSS vs. DHF	0.77 (0.42–1.41)	0.39	-	1.25 (0.46–3.98)	0.82	-	0.50 (0.20–1.23)	0.14	-

DHF: dengue hemorrhagic fever, DSS: dengue shock syndrome, OR: Odds ratio, 95% CI: Fisher exact 95% confidence intervals, P: Fisher's exact test, Pc: corrected P.

Certain alleles of the HLA-class I genes were previously found to be associated with DHF or DSS [1]. In a study of Thai patients, Stephens *et al.* found that HLA- A*0207 and HLA-B*51 were susceptible to both DHF and DSS [18]. It was also reported that HLA-A*29 and A*33 were protective against DHF and DSS in Cuban [33] and in Vietnamese patients, respectively [17]. The inconsistency of these HLA associations may be the result of the differences in ethnicity, geographic location or the diversity of the predominant virus during the study periods [2],[34].

The results of HLA class I association from the present study reproduced a previous Vietnamese study (309 cases and 251 controls) by Loke *et al.* in which HLA-A*24 association with DHF and DSS was found, with OR 1.54 and P value 0.021 [17]. Considering such reproducibility in different study periods, within the same ethnic group and in the same region, give us the confidence to assign an association of HLA-A*24 and DHF, DSS even though our P value just reached the significant level before Bonferoni correction.

### HLA-A*24 with Histidine at codon 70 was positively associated with DSS and DHF

It is known that HLA-A*24 alleles fall into at least two subtypes by the difference at codon 70 of alpha 1 domain, which together with codons 9, 45, 63, 66 and 67 composes peptide binding pocket B for P2 anchor position [35]. In the present study, there were 4 different A*24 alleles, A*2402, A*2403, A*2407 and A*2410 (Table 6). A*2407 is different from the others at codon 70 where histidine (CAC) is changed to glutamine (CAG). The frequencies of A*24 with histidine at codon 70 in DSS and DHF patients were significantly higher than that in the population background groups (HCMC 02-03 DSS: OR = 1.89, P = 0.008, DHF: OR = 1.75, P = 0.03; VL 02-03 DSS: OR = 1.70, P = 0.03, DHF OR = 1.46, P = 0.38; VL 04-05 DSS: OR = 2.09, P = 0.0075, DHF OR = 2.02, P = 0.038, Table 7). On the other hand, A*24 with glutamine at codon 70, A*2407 in our samples, did not show any significant difference in frequency (Table 6). Similar behavior of this allele was documented in Indian HIV study [36].

**Table 6:** Phenotype frequencies of HLA-A*24 subgroups

A*24		HCMC						VL									
		HCMC 02-03						VL 02-03				VL 04-05					
		DSS		DHF		Control		DSS		DHF		DSS		DHF		Control	
		No.	(%)	No.	(%)	No.	(%)	No.	(%)	No.	(%)	No.	(%)	No.	(%)	No.	(%)
1. A*24 with histidine at codon 70																	
	A*2402	46	(30.3)	34	(29.1)	43	(17.2)	53	(31.2)	8	(22.9)	34	(35.4)	18	(30.5)	38	(19.0)
	A*2403	0	(0.0)	1	(0.9)	2	(0.8)	1	(0.6)	1	(2.9)	1	(1.0)	2	(3.4)	1	(0.5)
	A*2410	2	(0.0)	0	(0.0)	4	(0.8)	0	(0.0)	1	(2.9)	0	(0.0)	1	(1.7)	4	(2.0)
	Total	48	(31.6)	35	(29.9)	49	(19.6)	54	(31.8)	10	(28.6)	35	(35.5)	21	(35.6)	43	(21.5)
2. A*24 with glutamine at codon 70																	
	A*2407	5	(3.3)	2	(1.7)	13	(5.2)	3	(1.8)	1	(2.9)	1	(1.0)	7	(11.9)	9	(4.5)

DHF: dengue hemorrhagic fever, DSS: dengue shock syndrome

**Table 7:** Association of HLA-A*24 with histidine at codon 70 and severe forms of DV infection

	HCMC		VL			
	HCMC 02-03		VL 02-03		VL 04-05	
	OR (95% CI)	P	OR (95% CI)	P	OR (95% CI)	P
DSS vs. control	1.89 (1.16–3.09)	0.008	1.70 (1.04–2.79)	0.03	2.09 (1.18–3.70)	0.0075
DHF vs. control	1.75 (1.02–2.98)	0.033	1.46 (0.58–3.44)	0.38	2.02 (1.01–3.95)	0.038

DHF: dengue hemorrhagic fever, DSS: dengue shock syndrome, OR: Odds ratio, 95% CI: Fisher exact 95% confidence intervals, P: Fisher's exact test. This analysis was perform after the confirmation of A^*^24 association, so that the correction of P value did not apply.

The association of HLA-A*24 in the VL 04-05 samples was stronger than in VL 02-03 and HCMC 02-03, possibly due to higher secondary infection rate in the VL 04-05 samples. Memory T cells might be important in the A*24 associated pathogenesis of DHF/DSS. HLA-A*24 is reported to be one of the potential restricting alleles for NS3 dengue viral protein and CTLs recognizing NS3 peptides coupled with HLA-A*24 were cross-reactive between serotypes because NS3 is highly conserved [37]. Two previous Vietnamese studies reported an identification of the DEN-2 NS3 556–564 peptide that was restricted by HLA-A*24 [17],[38], but further study is needed to clarify the role of A*24-restricted CD8 T-cells in the pathogenesis of DHF/DSS.

### HLA-DRB1*0901 was negatively associated with DSS

As shown in Table 4 and 5, the HLA-DRB1*0901 allele significantly decreased in frequency in DSS patients in VL 04-05 (OR = 0.37, P = 0.0018, Pc = 0.02) when compared to the control, mainly in secondary infection (OR = 0.35, P = 0.0025, Pc = 0.03), not in primary infection. This trend was also observed in the other 2 groups of samples but did not reach statistical significance (VL 02-03: OR 0.51, P = 0.026, Pc = 0.3; HCMC 02-03: OR = 0.89, P = 0.77).

Certain HLA class II alleles have been reported to confer a protective effect in infections of human immunodefiency virus (HIV), hepatitis C virus (HCV), and hepatitis B virus (HBV) [39]. In dengue virus infection, protective DRB1*07 and DRB1*04 alleles were recently reported. DRB1*07 was protective against DF, DHF and DSS and DRB1*04 was protective against secondary DF in Cuban population [34], DHF or DSS in Mexican population [40]. Possibly there is a common primary associated gene closely located to DRB1 locus, such as HLA-DQB1 and HLA-DRB4, for those alleles (DRB1*07 and DRB1*04) and Vietnamese DRB1*0901. One of the most frequent haplotypes among Oriental populations (including Kinh ethnic) is DRB1*0901-DQB1*030302. DQB1*030302 is also reported to be in disequilibrium with DRB1*0701 [41], however there was no reduced frequency of DRB1*0701 in DSS patients compared to controls in our data. Furthermore, haplotypes found in South America are DRB1*0701-DQB1*0201 and HLA-DRB1*0403-DQB1*0302, not DQB1*030302, which suggests that the association of these protective alleles could not be elucidated by a common DQB1 allele. The possibility of primary disease-protective association with DRB4*01 (encoding DR53 sero-specificity) remains to be examined because of DRB4*01 haplotypic association with DRB1*07, *04 and *0901 was found in all populations which have been studied [42], although neither DRB1*07 or DRB1*04 exhibited difference in frequency in our samples.

### Protective effect of HLA-DRB1*0901 against DSS development from DHF was evident when having DEN-2 infection

To see the effect of virus serotype on the HLA susceptibility, we analyzed the HLA alleles in the DEN-2-positive patients and those that were positive for DEN-1, DEN-3, or DEN-4 (DEN-2-negative). As shown in Table 8, HLA-DRB1*0901 frequency was significantly decreased in DSS compared with DHF among DEN-2- positive patients (OR = 0.13, P = 0.02). The effect of DRB1*0901 appeared to be DEN-2 specific because DEN-2-negative population did not show significant difference, although this may be due to the small sample size as Breslow-Day statistic for the uniformity of odds ratio did not reach significant level (P = 0.14).

**Table 8:** Phenotype frequency of HLA- DRB1*0901 allele in DHF or DSS patients with DEN-2 or other serotypes

Serotype	DSS	DHF	DSS vs. DHF	P
	No. (%)	No. (%)	OR (95% CI)	
DEN-2 (+)	n = 30	n = 20		
DRB1*0901 (+)	2 (6.7)	7 (35.0)	0.13 (0.01–0.86)	0.02
DRB1*0901 (−)	28 (93.3)	13 (65.0)		
DEN-2 (−)	n = 13	n = 24		
DRB1*0901 (+)	3 (23.1)	7 (29.2)	0.73 (0.10–4.22)	1
DRB1*0901 (−)	10 (76.9)	17 (70.8)		

DHF: dengue hemorrhagic fever, DSS: dengue shock syndrome,

DEN-2 (−): consisted of DEN-1, DEN-3, and DEN-4 as shown in Table 2.

OR: Odds ratio, 95%CI: Fisher exact 95% confidence intervals, P: Fisher's exact test.

The HLA-DRB1*0901 allele has also been reported at higher frequency in noncirrhotic HCV patients than in patients with cirrhosis [43],[44], suggesting protective effect of this allele against tissue destruction and disease progression. Here, when we focus on DEN-2 infection, a higher frequency of HLA-DRB1*0901 in DHF than in DSS patients was found. The protective effect of this allele against DSS development from DHF could be considered as a new finding in dengue infection. It is generally believed that DSS is preventable by intense clinical management, in fact a certain number of patients, however fall to shock in spite of the early efforts of physicians. Although the pathogenesis of DHF/DSS is still unclear, clinical studies have suggested that the host immune response shifted to Th2 dominant in most DSS cases [45]. Because HLA class II polymorphism can direct the antigen-specific T-cell response, it is probable that patients with HLA-DRB1*0901 may be prevented from the shift toward Th2 dominance. Further studies are needed to clarify this point.

In conclusion, our study confirmed the previous reported HLA association between HLA-A*24 and DHF or DSS and moreover showed that A*24 with Histidine at codon 70 to be susceptible to DSS and DHF, and HLA-DRB1*0901 to be protective against the development of DSS, particularly in patients with DEN-2 infection. This study represents another attempt to improve our understanding of the risk of HLA-class I for severe outcome of DV infection in the light of peptide anchor binding site and provides a novel evidence that HLA-class II may control disease severity (DHF to DSS) in DV infection.

## Supporting Information

Alternative Language Abstract S1 Translation of the Abstract into Japanese by Kenji Hirayama
(0.09 MB PDF)

Alternative Language Abstract S2 Translation of the Abstract into Vietnamese by Nguyen Thi Phuong Lan
(0.04 MB DOC)

Table S1 Phenotype frequencies of HLA-A, HLA-B and HLA-DRB1 alleles
(0.17 MB DOC)

## References

[1] Wagenaar JFP, Mairuhu ATA, van Gorp ECM (2004). Genetic influences on dengue virus infections.. Dengue Bulletin.

[2] Chaturvedi U, Nagar R, Shrivastava R (2006). Dengue and dengue haemorrhagic fever: implications of host genetics.. FEMS Immunol Med Mic.

[3] World Health Organization (1997). Dengue hemorrhagic fever: diagnosis, treatment, prevention, and control.. Geneva.

[4] Chaturvedi UC, Nagar R, Shrivastava R (2006). Macrophage and dengue virus: Friend or foe?. Indian J Med Res.

[5] World Health Organization (1999). Prevention and control of dengue and dengue hemorrhagic fever: comprehensive guidelines. WHO Regional publication, SEARO No. 29..

[6] Nguyen TKT (2004). Annual report of National program for dengue control in Vietnam..

[7] World Health Organization (2005). Dengue, dengue haemorrhagic fever and dengue shock syndrome in the context of the integrated management of childhood illness.. WHO/FCH/CAH/05.13.

[8] Halstead SB (2007). Dengue.. Lancet.

[9] Suharti C, van Gorp EC, Dolmans WM, Setiati TE, Hack CE (2003). Cytokine patterns during dengue shock syndrome.. Eur Cytokine Netw.

[10] Pang T, Cardosa MJ, Guzman MG (2007). Of cascades and perfect storms: the immunopathogenesis of dengue haemorrhagic fever-dengue shock syndrome (DHF/DSS).. Immunol Cell Biol.

[11] White NJ (1999). Variation in virulence of dengue virus.. Lancet.

[12] Loke H, Bethell D, Phuong CXT, Day N, White N (2002). Susceptibility to dengue hemorrhagic fever in Vietnam: evidence of an association with variation in the vitamin D receptor and Fc gamma receptor IIa genes.. Am J Trop Med Hyg.

[13] Sakuntabhai A, Turbpaiboon C, Casademont I, Chuansumrit A, Lowhnoo T (2005). A variant in the CD209 promoter is associated with severity of dengue disease.. Nat Genet.

[14] King NJ, Shrestha B, Kesson AM (2003). Immune modulation by flaviviruses.. Adv Virus Res.

[15] Spaulding AC, Kurane I, Ennis FA, Rothman AL (1999). Analysis of murine CD8+T-cell clones specific for the Dengue virus NS3 protein: flavivirus crossreactivity and influence of infecting serotype.. J Virol.

[16] Zivna I, Green S, Vaughn DW, Kalayanarooj S, Stephens HA (2002). T cell responses to an HLA-B*07-restricted epitope on the dengue NS3 protein correlate with disease severity.. J Immunol.

[17] Loke H, Bethell DB, Phuong CX, Dung M, Schneider J (2001). Strong HLA class I-restricted T cell responses in dengue hemorrhagic fever: a double-edged sword?. J Infect Dis.

[18] Stephens HA, Klaythong R, Sirikong M, Vaughn DW, Green S (2002). HLA-A and -B allele associations with secondary dengue virus infections correlate with disease severity and the infecting viral serotype in ethnic Thais.. Tissue Antigens.

[19] José G, Rigau-Pérez (2006). Severe dengue: the need for new case definitions.. Lancet Infect Dis.

[20] Innis BL, Nisalak A, Nimmannitva S, Kusalerdchariya S, Chongswasdi V (1989). An enzyme-linked immunosorbent assay to characterize dengue infections where dengue and Japanese encephalitis co-circulate.. Am J Trop Med Hyg.

[21] Gubler DJ, Kuno G, Sather GE, Valez M, Oliver A (1984). Mosquito cell cultures and specific monoclonal antibodies in surveillance for dengue viruses.. Am J Trop Med Hyg.

[22] Lanciotti RS, Calisher CH, Gubler DJ, Chang GJ, Vorndam AV (1992). Rapid detection and typing of dengue viruses from clinical samples by using reverse transcriptase-polymerase chain reaction.. J Clin Microbiol.

[23] Charron D, Ishikawa Y, Tanaka H, Semana G, Tiercy JM, Fan L, Blasczyk R (1997). 12^th^ International Histocompatibility Workshop and Conference Proceedings: Genetic Diversity of HLA: Functional and Medical Implications.. 12th AHS#2 report 1.

[24] Butt N, Abbassi A, Munir SM, Ahmad SM, Sheikh QH (2008). Haematological and Biochemical Indicators for the Early Diagnosis of Dengue Viral Infection.. J Coll Physicians Surg Pak.

[25] Faridi MM, Aggarwal A, Kumar M, Sarafrazul A (2008). Clinical and biochemical profile of dengue haemorrhagic fever in children in Delhi.. Trop Doct.

[26] Díaz-Quijano FA, Martínez-Vega RA, Villar-Centeno LA (2005). Early indicators of severity in dengue virus infection.. Enferm Infecc Microbiol Clin.

[27] Pham TB, Nguyen TH, Vu TQ, Nguyen TL, Malvy D (2007). Predictive factors of dengue shock syndrome at the Children hospital No.1, Ho Chi Minh City, Vietnam.. Bull Soc Pathol Exot.

[28] Chao DY, Lin TH, Hwang KP, Liu CC, King CC (2004). 1998 dengue hemorrhagic fever in Taiwan.. Emer Infect Dis.

[29] Sriprom M, Pongsumpun P, Yoksan S, Barbazan P, Gonzaler JP (2003). Dengue hemorrhagic fever in Thailand, 1998-2003: Primary or secondary infection.. Dengue Bull.

[30] Halstead SB, Lan NT, Myint TT, Shwe TN, Nisalak A Dengue Hemorrhagic Fever in Infants: Research Opportunities Ignored.. Emerg Infect Dis.

[31] Islam MA, Ahmed MU, Begum N, Chowdhury NA, Khan AH (2006). Molecular characterization and clinical evaluation of dengue outbreak in 2002 in Bangladesh.. Jpn J Infect Dis.

[32] Vaughn DW, Green S, Kalayanarooj S, Innis BL, Nimmannitya S (2000). Dengue viremia titer, antibody response pattern, and virus serotype correlate with disease severity.. J Infect Dis.

[33] Paradoa Perez ML, Trujillo Y, Basanta P (1987). Association of dengue hemorrhagic fever with the HLA system.. Haematol.

[34] Beatriz Sierra, Roberto Alegre, Pérez AnaB, Gissel García (2007). HLA-A, -B, -C, and -DRB1 allele frequencies in Cuban individuals with antecedents of dengue 2 disease: Advantages of the Cuban population for HLA studies of dengue virus infection.. Human Immunol.

[35] Sidney J, Peters B, Frahm N, Brander C, Settle A (2008). HLA class I supertypes: a revised and updated classification.. BMC Immunol.

[36] Umapathy S, Pawar A, Ghosh K (2007). Specific human leukocyte antigen alleles associated with HIV-1 infection in an Indian population.. J Acquir Immune Defic Syndr.

[37] Mathew A, Kurane I, Rothman AL, Zeng LL, Brinton MA (1996). Dominant recognition by human CD8-cytotoxic T lymphocytes of dengue virus nonstructural proteins NS3 and NS1.2a.. J Clin Invest.

[38] Simmons Cp, Dong T, Chau NV, Dung NT, Chau TN (2005). Early T-cell responses to dengue virus epitopes in Vietnamese adults with secondary dengue virus infections.. J Virol.

[39] Maureen PM, Carrington M (2005). Immunogenetics of viral infections.. Curr Opin Immunol.

[40] LaFleur C, Granados J, Vargas-Alarcon G, Ruiz-Morales J, Villarreal-Garza C (2002). HLA-DR antigen frequencies in Mexican patients with dengue virus infection: HLA-DR4 as a possible genetic resistance factor for dengue hemorrhagic fever.. Hum Immunol.

[41] Charron D, Clayton J, Lonjou C (1997). 12th International Histocompatibility Workshop and Conference Proceedings: Genetic Diversity of HLA: Functional and Medical Implications.. Whittle D. Allele and haplotype frequencies for HLA loci in various ethnic groups.

[42] Burdett L, Smith K, Tu B, Guiterrez M, Buck K, Maiers M (2003). DRB-DQB1 diversity in the analysis of 4727 donors tested by SBT.. Human Immunology.

[43] Tokushige K, Tsuchiya N, Hasegawa K, Hashimoto E, Yamauchi K (2003). Influence of TNF gene polymorphism and HLA-DRB1 haplotype in Japanese patients with chronic liver disease caused by HCV.. Am J Gastroenterol.

[44] Aikawa T, Kojima M, Onishi H, Tamura R, Fukuda S (1996). HLA DRB1 and DQB1 alleles and haplotypes influencing the progression of hepatic C.. J Med Virol.

[45] Chaturvedi UC, Agarwal R, Elbishbish EA, Mustafa AS (2000). Cytokine cascade in dengue hemorrhagic fever: implications for pathogenesis.. FEMS Immunol Med Mic.

