# Supplementary material for: Protective and Enhancing HLA Alleles, HLA-DRB1*0901 and HLA-A*24, for Severe Forms of Dengue Virus Infection, Dengue Hemorrhagic Fever and Dengue Shock Syndrome
Source: PLoS Negl Trop Dis. 2008 Oct 1;2(10):e304. doi: 10.1371/journal.pntd.0000304 (PMC2553281; doi:10.1371/journal.pntd.0000304)
Supplement: Alternative Language Abstract S1 — Translation of the Abstract into Japanese by Kenji Hirayama (0.09 MB PDF) [file pntd.0000304.s001.pdf]

Japanese Translation

デングウイルス感染症の重症化（デング出血熱、デングショック症候群）に対して HLA-DRB1*0901 は抵抗性、HLA-A*24 は感受性を示した

要約

*背景：*デングウイルス感染症は熱帯地域で流行する蚊媒介性疾患の中でも重要な感染症となっている。近年南部ベトナムにおいては、重症型であるデング出血熱あるいはデングショック症候群は小児の死亡原因のトップになっている。感染後の重症化の機序は不明だが、特徴的な病態の形成にはヒト宿主のＴ細胞性の免疫応答が重要な役割を果たしていると考えられている。

方法と主要な結果： デングウイルスに対するＴ細胞応答を制御する特徴的な HLA 対立遺伝子を解析するために、病院ベースの患者—対照研究を南部ベトナムのホーチミン市とビンロン県の二つの病院で行った。WHO の診断基準を基に診断を行い、全部で出血熱患者 211 名、ショック症候群患者 418 名、の末梢血 DNA を採取し、またホーチミン市 250 名、ビンロン県 200 名の健常小児を対照となる背景集団として用い、HLA-A, B, DRB1 遺伝子座の対立遺伝子の解析を行った。その結果、HLA-A*24 の頻度が重症型で有意に上昇していることが明らかになった。さらに HLA-A*24 の亜型であり 70 番目のコドンがヒスチジンに置換されている A*2402/03/10 がとりわけ著明に上昇していることが判明した

(HCMC 02-03 DSS: OR = 1.89, P = 0.008, DHF: OR = 1.75, P = 0.03; VL 02-03 DSS: OR = 1.70, P = 0.03, DHF OR = 1.46, P = 0.38; VL 04-05 DSS: OR = 2.09, P = 0.0075, DHF OR = 2.02, P = 0.038)。

これに対して、HLA-DRB1*0901 の頻度はビンロン県の再感染グループのショック症候群で有意に減少していた (OR = 0.35, P = 0.0025, Pc = 0.03)。この傾向はデングウイルス 2 型に感染した患者ではより明白で、重症型のなかでもショック症候群でその頻度が出血熱と比較して有意に減少していた（p<0.02）。

結論：本研究によりデングウイルス感染症の重症化における HLA クラスⅠ分子の抗原提示分子としての重要性がより強く示唆され、また HLA クラスⅡ遺伝子の抵抗性への関与が新たに示された。
