# Supplementary material for: Protective and Enhancing HLA Alleles, HLA-DRB1*0901 and HLA-A*24, for Severe Forms of Dengue Virus Infection, Dengue Hemorrhagic Fever and Dengue Shock Syndrome
Source: PLoS Negl Trop Dis. 2008 Oct 1;2(10):e304. doi: 10.1371/journal.pntd.0000304 (PMC2553281; doi:10.1371/journal.pntd.0000304)
Supplement: Alternative Language Abstract S2 — Translation of the Abstract into Vietnamese by Nguyen Thi Phuong Lan (0.04 MB DOC) [file pntd.0000304.s002.doc]

Vietnamese translation

**Vai trò của HLA-DRB1*0901 and HLA-A*24 trong các thể bệnh nặng của nhiễm vi-rút dengue: sốt xuất huyết dengue và hội chứng sốc dengue**

**Tóm tắt**

***Đặt vấn đề*:** Nhiễm vi-rút dengue là một trong những bệnh lan truyền do muỗi quan trọng ở các nước nhiệt đới. Các dạng bệnh nặng như sốt xuất huyết dengue (SXHD) và hội chứng sốc dengue (HCSD) gần đây đã trở thành một trong những nguyên nhân hàng đầu gây tử vong cho trẻ em ở khu vực phía nam Việt nam. Đáp ứng miễn dịch qua tế bào lympho T được đánh giá có vai trò quan trọng trong việc bảo vệ/ sinh bệnh học của SXHD và HCSD.

***Phương pháp nghiên cứu/ kết qủa:*** Để xác định alen HLA kiểm soát miễn dịch tế bào T chống lại vi-rút dengue, chúng tôi đã tiến hành một nghiên cứu bệnh-chứng thực hiện tại hai bệnh viện ở khu vực phía nam Việt nam (bệnh viện Nhi Đồng 2 thành phố Hồ Chí Minh (Tp.HCM) và bệnh viện tỉnh Vĩnh Long (VL)), từ năm 2002-2005. Nhóm bệnh gồm 211 bệnh nhân SXHD và 418 bệnh nhân HCSD chẩn đoán theo tiêu chuẩn phân độ của Tổ chức Y tế thế giới, được phân tích về các alen HLA-A, -B và - DRB1. Nhóm chứng gồm 450 trẻ em dân tộc Kinh, khỏe mạnh từ Tp.HCM (250) và từ VL (200) cũng được phân tích. Trong HLA lớp I, tần suất của HLA-A*24 có chiều hướng gia tăng ở cả hai nhóm SXHD và HCSD so với nhóm chứng, phù hợp với kết quả của một nghiên cứu trước đây. Phân tích dựa trên tính đặc hiệu của vị trí kết nối quan trọng giữa pép-tit và phân tử HLA, HLA-A*24 có Histidine ở axit amin 70 (A*2402/03/10) của các nhóm SXHD và HCSD có tần suất cao hơn nhóm chứng một cách có ý nghĩa thống kê. (Tp.HCM 02-03 HCSD: OR = 1.89, P = 0.008, SXHD: OR = 1.75, P = 0.033; VL 02-03 HCSD: OR = 1.70, P = 0.03, SXHD: OR = 1.46, P = 0.38; VL 04-05 HCSD: OR = 2.09, P = 0.0075, SXHD: OR = 2.02, P = 0.038). Trong HLA lớp II, tần suất của HLA-DRB1*0901 giảm một cách có ý nghĩa thống kê ở nhóm HCSD nhiễm dengue thứ phát của VL 04-05 khi so với nhóm chứng (OR = 0.35, P = 0.0025, Pc = 0.03). Đặc biệt hơn nữa, tần suất của HLA-DRB1*0901 giảm một cách có ý nghĩa thống kê ở nhóm HCSD so với nhóm SXHD trong nhiễm vi-rút DEN-2 (P = 0.02).

***Kết luận:*** Nghiên cứu này giúp chúng ta hiểu thêm về nguy cơ phát triển bệnh nặng của HLA lớp I trong nhiễm vi-rút dengue dựa trên đặc tính của vị trí kết nối giữa pép-tit của vi-rút và phân tử HLA, đồng thời đưa ra chứng cứ mới cho rằng HLA lớp II có thể kiểm soát sự trầm trọng (SXHD sang HCSD) của bệnh này.
