# Supplementary material for: Protective and Enhancing HLA Alleles, HLA-DRB1*0901 and HLA-A*24, for Severe Forms of Dengue Virus Infection, Dengue Hemorrhagic Fever and Dengue Shock Syndrome
Source: PLoS Negl Trop Dis. 2008 Oct 1;2(10):e304. doi: 10.1371/journal.pntd.0000304 (PMC2553281; doi:10.1371/journal.pntd.0000304)
Supplement: Table S1 — Phenotype frequencies of HLA-A, HLA-B and HLA-DRB1 alleles (0.17 MB DOC) [file pntd.0000304.s003.doc]

Table S1. Phenotype frequencies of HLA-A, HLA-B and HLA-DRB1 alleles

|  | HCMC |  |  |  |  |  |  |  | VL |  |  |  |  |  |  |  |  |  |  |  |
| --- | --- | --- | --- | --- | --- | --- | --- | --- | --- | --- | --- | --- | --- | --- | --- | --- | --- | --- | --- | --- |
|  | ND02-03 | |  |  |  |  |  |  | VL02-03 | |  |  |  | VL04-05 | |  |  |  |  | |
|  | DSS | | DHF | |  | Control | |  | DSS | | DHF | |  | DSS | | DHF | |  | Control | |
|  | n = 152 | | n = 117 | |  | n = 250 | |  | n = 170 | | n = 35 | |  | n = 96 | | n = 59 | |  | n = 200 | |
| Phenotype | No. | (%) | No. | (%) |  | No. | (%) |  | No. | (%) | No. | (%) |  | No. | (%) | No. | (%) |  | No. | (%) |
| HLA-A |  |  |  |  |  |  |  |  |  |  |  |  |  |  |  |  |  |  |  |  |
| *01 | 8 | (5.3) | 9 | (7.7) |  | 16 | (6.4) |  | 9 | (5.3) | 3 | (8.6) |  | 6 | (6.3) | 3 | (5.1) |  | 10 | (5.0) |
| *02 | 62 | (40.8) | 40 | (34.2) |  | 122 | (48.8) |  | 67 | (39.4) | 11 | (31.4) |  | 43 | (44.8) | 22 | (37.3) |  | 80 | (40.0) |
| *11 | 59 | (38.8) | 58 | (49.6) |  | 116 | (46.4) |  | 87 | (51.2) | 20 | (57.1) |  | 44 | (45.8) | 25 | (42.4) |  | 95 | (47.5) |
| *24 | 53 | (34.9) | 36 | (30.8) |  | 62 | (24.8) |  | 57 | (33.5) | 11 | (31.4) |  | 36 | (37.5) | 28 | (47.5) |  | 50 | (25.0) |
| *26 | 10 | (6.6) | 7 | (6.0) |  | 9 | (3.6) |  | 4 | (2.4) | 0 | (0.0) |  | 5 | (5.2) | 1 | (1.7) |  | 9 | (4.5) |
| *29 | 18 | (11.8) | 16 | (13.7) |  | 33 | (13.2) |  | 25 | (14.7) | 4 | (11.4) |  | 11 | (11.5) | 7 | (11.9) |  | 31 | (15.5) |
| *30 | 6 | (3.9) | 7 | (6.0) |  | 8 | (3.2) |  | 4 | (2.4) | 1 | (2.9) |  | 1 | (1.0) | 1 | (1.7) |  | 7 | (3.5) |
| *33 | 42 | (27.6) | 23 | (19.7) |  | 54 | (21.6) |  | 41 | (24.1) | 12 | (34.3) |  | 29 | (30.2) | 11 | (18.6) |  | 45 | (22.5) |
| HLA-B |  |  |  |  |  |  |  |  |  |  |  |  |  |  |  |  |  |  |  |  |
| *07 | 24 | (15.8) | 23 | (19.7) |  | 41 | (16.4) |  | 25 | (14.7) | 6 | (17.1) |  | 17 | (17.7) | 10 | (16.9) |  | 28 | (14.0) |
| *13 | 21 | (18.0) | 21 | (17.9) |  | 23 | (9.2) |  | 18 | (10.6) | 3 | (8.6) |  | 9 | (9.4) | 6 | (10.2) |  | 18 | (9.0) |
| *15 | 54 | (35.5) | 57 | (48.7) |  | 109 | (43.6) |  | 69 | (40.6) | 15 | (42.9) |  | 33 | (34.4) | 28 | (47.5) |  | 87 | (43.5) |
| *27 | 5 | (3.3) | 2 | (1.7) |  | 21 | (8.4) |  | 6 | (3.5) | 0 | (0.0) |  | 3 | (3.1) | 4 | (6.8) |  | 13 | (6.5) |
| *35 | 14 | (9.2) | 9 | (7.7) |  | 14 | (5.6) |  | 12 | (7.1) | 3 | (8.6) |  | 8 | (8.3) | 4 | (6.8) |  | 14 | (7.0) |
| *38 | 31 | (20.4) | 13 | (11.1) |  | 40 | (16.0) |  | 28 | (16.5) | 3 | (8.6) |  | 10 | (10.4) | 5 | (8.5) |  | 32 | (16.0) |
| *40 | 27 | (17.8) | 14 | (12.0) |  | 25 | (10.0) |  | 24 | (14.1) | 4 | (11.4) |  | 11 | (11.5) | 10 | (16.9) |  | 37 | (18.5) |
| *44 | 8 | (5.3) | 7 | (6.0) |  | 19 | (7.6) |  | 9 | (5.3) | 2 | (5.7) |  | 12 | (12.5) | 1 | (1.7) |  | 9 | (4.5) |
| *46 | 27 | (17.8) | 21 | (17.9) |  | 55 | (22.0) |  | 33 | (19.4) | 7 | (20.0) |  | 16 | (16.7) | 14 | (23.7) |  | 40 | (20.0) |
| *51 | 6 | (3.9) | 10 | (8.5) |  | 14 | (5.6) |  | 18 | (10.6) | 3 | (8.6) |  | 11 | (11.5) | 4 | (6.8) |  | 12 | (6.0) |
| *55 | 5 | (3.3) | 6 | (5.1) |  | 18 | (7.2) |  | 7 | (4.1) | 1 | (2.9) |  | 6 | (6.3) | 4 | (6.8) |  | 13 | (6.5) |
| *57 | 9 | (5.9) | 4 | (3.4) |  | 11 | (4.4) |  | 10 | (5.9) | 3 | (8.6) |  | 7 | (7.3) | 5 | (8.5) |  | 12 | (6.0) |
| *58 | 26 | (17.1) | 18 | (15.4) |  | 30 | (12.0) |  | 24 | (14.1) | 8 | (22.8) |  | 14 | (14.5) | 7 | (11.9) |  | 28 | (14.0) |
| HLA-DRB1 |  |  |  |  |  |  |  |  |  |  |  |  |  |  |  |  |  |  |  |  |
| *0301 | 20 | (13.2) | 11 | (9.4) |  | 27 | (10.8) |  | 18 | (10.6) | 9 | (25.7) |  | 10 | (10.4) | 3 | (5.1) |  | 20 | (10.0) |
| *0403 | 13 | (8.6) | 7 | (6.0) |  | 10 | (4.0) |  | 10 | (5.9) | 2 | (5.7) |  | 4 | (4.2) | 4 | (6.8) |  | 10 | (5.0) |
| *0405 | 14 | (9.2) | 10 | (8.5) |  | 30 | (12.0) |  | 22 | (12.9) | 3 | (8.6) |  | 12 | (12.5) | 9 | (15.3) |  | 24 | (12.0) |
| *0701 | 22 | (14.5) | 15 | (12.8) |  | 30 | (12.0) |  | 19 | (11.2) | 7 | (20.0) |  | 20 | (20.8) | 7 | (11.9) |  | 28 | (14.0) |
| *0803 | 12 | (7.9) | 16 | (13.7) |  | 14 | (5.6) |  | 16 | (9.4) | 1 | (2.9) |  | 8 | (8.3) | 3 | (5.1) |  | 12 | (6.0) |
| *0901 | 33 | (21.7) | 31 | (26.5) |  | 61 | (24.4) |  | 35 | (20.6) | 6 | (17.1) |  | 14 | (14.6) | 15 | (25.4) |  | 63 | (31.5) |
| *1001 | 20 | (13.2) | 16 | (13.7) |  | 31 | (12.4) |  | 18 | (10.6) | 5 | (14.3) |  | 14 | (14.6) | 8 | (13.6) |  | 15 | (7.5) |
| *1202 | 74 | (48.7) | 47 | (40.2) |  | 121 | (48.4) |  | 78 | (45.9) | 21 | (60.0) |  | 40 | (41.7) | 35 | (59.3) |  | 99 | (49.5) |
| *1401 | 7 | (4.6) | 5 | (4.3) |  | 17 | (6.8) |  | 12 | (7.1) | 2 | (5.7) |  | 6 | (6.3) | 4 | (6.8) |  | 11 | (5.5) |
| *1501 | 13 | (8.6) | 12 | (10.3) |  | 11 | (4.4) |  | 12 | (7.1) | 1 | (2.9) |  | 9 | (9.4) | 5 | (8.5) |  | 10 | (5.0) |
| *1502 | 27 | (17.8) | 17 | (14.5) |  | 43 | (17.2) |  | 32 | (18.8) | 1 | (2.9) |  | 18 | (18.8) | 5 | (8.5) |  | 35 | (17.5) |
| *1602 | 5 | (3.3) | 6 | (5.1) |  | 9 | (3.6) |  | 10 | (5.9) | 2 | (5.7) |  | 5 | (5.2) | 4 | (6.8) |  | 8 | (4.0) |

DHF: dengue hemorrhagic fever, DSS: dengue shock syndrome.
